# Factors Predisposing to The Formation of Degenerative Spondylolisthesis—A Narrative Review

**DOI:** 10.3390/medicina59081430

**Published:** 2023-08-07

**Authors:** Marek Mazurek, Bartłomiej Kulesza, Natalia Gołębiowska, Bartłomiej Tyzo, Krzysztof Kura, Dariusz Szczepanek

**Affiliations:** 1 Department of Neurosurgery and Pediatric Neurosurgery, Medical University of Lublin, 20-954 Lublin, Poland; 2 Department of Medical Chemistry, Medical University of Lublin, 20-093 Lublin, Poland; 3 Department of Neurosurgery and Spine Surgery, Regional Hospital in Kielce, 25-736 Kielce, Poland

**Keywords:** lumbar spine, degenerative spondylolisthesis, risk factors, diagnostic radiology, degenerative disease, sex hormones

## Abstract

The relationship between various factors predisposing to the formation of spondylolisthesis, including degenerative spondylolisthesis, has been analyzed by many authors. However, not all observations are consistent. In this review, we identified factors whose impact on the prevalence of spondylolisthesis was most often mentioned in the literature. These included gender, age, bone mineral density, ethnic origin, and oophorectomy. The results were inclusive in terms of physical activity, pregnancy status, and use of hormone replacement therapy. Associations between diabetes and smoking were very poorly marked. The literature so far has identified a number of factors significantly affecting the incidence of degenerative spondylolisthesis. These include age, gender, body weight, ethnic origin, bone mineral density, and hormonal balance. Radiological parameters, which include iliac crest, pelvic tilt, pelvic incidence, sacral slope, and lumbar lordosis, may also be of great importance for assessing changes in the occurrence and progression. However, the authors do not agree on the real significance of individual factors. The aim of this review was to identify the factors predisposing to the formation of degenerative spondylolisthesis, the importance of which has been suggested in the current literature. The systematization of knowledge in this field can allow a more accurate adjustment of the treatment plan for each patient affected by this condition.

## 1. Introduction

The human spine is a very complex structure whose individual elements play a key role in the mechanisms maintaining vertical body posture and movement. Unfortunately, due to the very heavy loads that affect the spine, we often deal with the formation of various types of degenerative changes. One of the diseases in this group is degenerative spondylolisthesis. Spondylolisthesis is defined as the displacement of at least one vertebra in relation to the vertebra (or sacrum) below it. The most common type of spondylolisthesis is a forward displacement of a vertebra (anterolisthesis), but retrolisthesis is also a relatively common phenomenon [1,2]. It is estimated that in the world, this problem affects from 3% to even 30% of the population depending on gender and ethnicity [3,4,5,6,7,8,9,10,11]. Congenital abnormalities, previous injuries, surgical procedures, concomitant diseases, and degenerative processes can lead to the formation of a slip [12]. Due to the aging of society, degenerative processes pose an increasing challenge to modern medicine. 

Degenerative spondylolisthesis (DS) is a type of lesion arising from the loss of spine stability and chronic degenerative changes [12,13]. It is characteristic of older people (Adult Spondylolisthesis in the Low Back, American Academy of Orthopedic Surgeons). As the population ages, older adults pose an increasingly serious problem for modern spine surgery. For this reason, this review focuses on degenerative spondylolisthesis.

The purpose of this review is to thoroughly assess and summarize the existing knowledge about socio-demographic and environmental factors affecting the prevalence of degenerative spondylolisthesis in the population. To the best of our knowledge, this is the only review that presents all the described risk factors for the development and progression of degenerative spondylolisthesis. In order to conduct such a thorough analysis of all the risk factors for the development of degenerative spondylolisthesis, in this review, we considered scientific papers whose age and quality do not meet contemporary standards of the scientific literature. For this reason, the proper preparation of a systemic review in the traditional form was very difficult. Therefore, the following review is intended as a narrative review to highlight the conclusions of these studies and contrast them with more contemporary reports. The characteristics of studies included in the analysis are presented in Table 1.

### Etiology

The etiology of degenerative spondylolisthesis is associated with the degenerative changes in individual elements of the spinal column, which, as the changes progress, lose the ability to resist the stresses affecting them, gradually leading to intersegmental instability at the lumbar motion segment [34,35,36,37]. The importance of the three main disorders that result in the formation of a slip is emphasized as follows:

- Arthritis and orientation of the facet joint favoring increased mobility;
- Disturbances in the functions of the ligament system stabilizing the column;
- Ineffective functioning of postural muscles [35,36,37,38,39,40,41,42,43,44].

Some authors believe that intervertebral disc degeneration may initiate spondylolisthesis [45,46]. Interestingly, in an analysis of CT discograms in patients with DS, Ito et al. indicated a number of degenerative changes in discs predisposing to the formation of a slip [47]. In 66% of cases, degenerative spondylolisthesis affects only one level of the spine, most often L4-L5 [10,37,48]. This may be due to the high value of the pedicle–facet angle [49], the coronal orientation of the L5-S1 facets, and the sagittal-placed facet joint [15,38,40,50,51,52,53,54,55]. An additional factor predisposing to the formation of a slip at this level may be the weakening of the iliopsoas ligaments. Proceedings on the condition of the ligament apparatus in the etiology of degenerative spondylolisthesis can be found in several studies [17,56,57,58]. The paraspinal muscles and other muscle groups, such as the deep abdominal muscles, are involved in maintaining the stability of the spine. Weakening of the multifidus, transverse abdominis, or pelvic floor muscles are other factors predisposing to the formation of degenerative spondylolisthesis [59,60,61,62,63].

The forces acting on the spinal column system are closely related to the role of the population and environmental factors in the formation of this type of pathology.

## 2. Literature Search for Evidence

The following databases were used to search the literature: PubMed, Google Scholar, BMC, Embase, and SCOPUS search engines. Additionally, the papers identified by searching bibliographies and those suggested by experts during the preparation of this review were included in the analysis. All included papers were written in English. 

The database search was carried out between March 2020 and February 2022.

Studies related to trauma, iatrogenic lesions, and isthmic spondylolisthesis were excluded. Studies on people under the age of 18 and on patients diagnosed with cancer diseases were also excluded. In addition, observations focusing only on radiological factors affecting the spread of a slip were also excluded from this review.

## 3. Risk Factors

### 3.1. Physical Effort and Professional Activity

The human spine is a very complex mechanical system that enables efficient functioning under physiological loads. However, when its elements are subjected to excessive forces, degenerative changes occur [64,65,66]. This phenomenon may result from both professional work and recreational activity. Many authors have investigated the relationship between excessive loads acting on the spinal column with a predisposition to degenerative slip formation. For example, Mariconda et al. analyzed 120 cases of back pain patients with spondylolisthesis. They showed a greater prevalence of slips among patients who actively played sports [15]. A similar relationship was demonstrated by Denard et al. in their observations on men over 65. They used the Physical Activity Scale for the Elderly (PASE) to measure daily physical activity. The authors noted that the prevalence of spondylolisthesis was greater among men with higher PASE scores. A detailed analysis showed a greater prevalence in people who spend their free time being active (43% vs. 31%) [9]. Many authors also analyzed the professional activity of patients. For example, a study by Mariconda et al. suggested a relationship between high physical effort and lifetime work exposure on the prevalence of spondylolisthesis [15]. A more detailed analysis of activity type was performed by Matsunga et al. They analyzed the progression of degenerative changes in 40 patients with spondylolisthesis. Their results showed that occupations that require repetitive anterior flexion, such as farming or fishing, are a risk factor for slide progression. In the group of people with intensified changes, as many as 75% of patients performed this type of activity, while among people without progression, this percentage was only 10% [39]. This issue was also studied by Ischimoto et al. using observations of Japanese adults from the Wakayama Spine Study. The authors demonstrated that among people under the age of 75, the most important risk factor for the occurrence of slip was work in the agricultural/fishing industries, which was associated with a 3 times greater risk of this type of change [33]. Similar conclusions were shown in the observations of other authors [67,68,69,70]. Interestingly, not only is occupational exposure to greater physical effort important but also the long-term operation in a certain position of the body. This applies, among others, to professional drivers who spend many hours in cars. Chen et al. analyzed the Taxi Drivers’ Health Study (TDHS) population of professional drivers in Taipei for spondylolisthesis. Out of all the examined persons, the presence of slip was detected in 40 cases (3.2%). The authors showed that the length of time working as a taxi driver significantly affected the risk of spondylolisthesis. In the group of drivers working in the profession for up to 5 years, the frequency of spondylolisthesis was only 1.1%, while for those working 6–15 years, this value was more than twice as high. Among those working for over 15 years, 7.1% were affected by a slip. Interestingly, the authors did not show the impact of physical activities (lifting tasks, bending/twisting) at work on the occurrence of this type of change [6]. The impact of a forced body position was also noted in the observations by Mariconda et al. In their study group, occupational driving was the only factor associated with a greater degree of vertebral slip. Moreover, they demonstrated the influence of prolonged occupational sitting on the prevalence of spondylolisthesis [15]. Ishimoto et al. also noted a relationship between slip and time spent driving a car. In this case, patients who reported occupational driving for ≥4 h/day were more than two times more likely to develop spondylolisthesis. The situation was similar in the group of people aged ≥75 years who reported sedentary work for ≥2 h/day [33]. Additionally, some authors suggested a role of whole-body vibration, to which vehicle drivers were exposed as the main mechanism influencing the pathogenesis of a slip [71,72,73]. On the other hand, data from some centers seem to contradict this thesis. A study by Jacobsen et al. did not suggest any relationship between professional activity and the prevalence of spondylolisthesis [7]. Similar results regarding professional activity were seen in the prospective population-based study by Wang et al. They showed no effect of a long-term occupation requiring physical effort on the prevalence of spondylolisthesis or on the progression of spondylolisthesis in both women and men. Moreover, among women, lower physical activity was a significant factor associated with spondylolisthesis progression [74]. He et al. analyzed a similar group of patients and showed that the level of physical activity measured with PASE was significantly higher in the male group without a slip compared to the male group with a slip. They suggested the role of daily exercise in preventing spondylolisthesis [27]. This was also consistent with the observations of Ishimoto et al. These authors showed that people under 75 years of age were less likely to develop spondylolisthesis if they climbed up stairs and slopes (≥1 h/day). Similarly, in the older group (≥75 years), walks of ≥3 km/day were a protective factor against a slip, in contrast to the observations of Denard et al. [33]. It is worth noting that in the study by Denard et al., daily walking was a risk factor for a listhesis [9]. The above data indicate an ambiguous role of physical activity in the predisposition to the formation of slip. The role of physical activity probably depends on the intensity of the effort and its characteristics. Certain exercises and types of physical activity may improve the natural mechanisms underlying protection against instability, such as paraspinal muscle tension, while others will lead to overloading and exacerbating degenerative changes. However, a full understanding of this issue requires further, more detailed observations.

### 3.2. Body Mass

Overloading the spinal column does not have to be caused by excessive physical activity. It may also be the result of excessive body weight. It has been demonstrated repeatedly that with an increase in body mass index (BMI), the axial load on the discs and facet joints, as well as compressive force acting on the vertebrae, increases. Chronic exposure to this type of stressor may contribute to increased intersegmental mobility, anterior displacement of the trunk, and an enhanced risk of degenerative spondylolisthesis [6,7,24,26,31,57,75]. In the aforementioned work, Jacobsen et al. drew attention to another feature that may affect the prevalence of degenerative spondylolisthesis: the weight of patients. The authors noted a more frequent occurrence of slips in individuals with higher BMI values. Interestingly, this only concerned women. In the case of men, a similar relationship was not observed [7]. In several other studies on spondylolisthesis, the authors also emphasized the role of body weight in the etiology of such changes. A study carried out by He et al. on the elderly population in China showed that a larger BMI is a risk factor for slippage among both women and men [27]. Chen et al. obtained similar conclusions by examining the prevalence of slips among professional drivers. In their study, the relationship concerned both sexes. However, it should be noted that the majority of respondents consisted of men [6]. The influence of BMI on the prevalence of degenerative spondylolisthesis was also observed by Schuller et al. In their study, the mean body mass index value for the group of people with spondylolisthesis was 28.2, while for the control group, it was only 24.8. Moreover, the authors showed that as many as 71.4% of patients with a slip are overweight or obese. This problem affected 50.6% of the control group. Schuller et al. also noted the relationship between high BMI values and the sagittal facet joint orientation at the L4-L5 level [24]. It has been repeatedly suggested that this type of setting is a risk factor for degenerative spondylolisthesis [76,77,78,79]. The existence of an analog relationship between the prevalence of spondylolisthesis and BMI values was also noted by other authors [26,31,74,80]. Moreover, this factor may also influence the effects of applied treatments. Ebstein et al. found high body weight as the main cause of surgery failures in patients with degenerative slips [81]. Rihn et al. analyzed the treatment results of patients with degenerative spondylolisthesis depending on body weight. The authors divided patients into two groups: obese (with BMI > 30) and non-obese (with BMI < 30). Then, the groups were examined using widely recognized scales and questionnaires to assess the intensity of their symptoms and quality of life after treatment. In non-operated patients, obesity had worse results in the ODI and the SF-36 physical function. An analysis of the history of surgically treated patients showed that BMI > 30 was associated with twice the risk of reoperation (20% vs. 11%) and a 5 times higher infection rate (5% vs. 1%). In addition, they achieved worse results in the SF36 physical function score. It is worth mentioning that in the same study, the effect of obesity on the treatment of patients with lumbar stenosis was also examined, but no relationship was found in the case of operated patients [82]. On the other hand, the results obtained by some researchers did not show a significant relationship between BMI values and the prevalence of spondylolisthesis [9,25,32,57,83,84]. Surprising observations were made by Kauppila et al. The authors showed no correlation between the prevalence of spondylolisthesis and the weight of patients in the case of single-level changes. However, the subjects with two or more slipped vertebrae were characterized by a lower body weight compared to subjects without slips (63 ± 9.6 kg vs. 69 ± 13 kg) [18].

### 3.3. Anatomical Features

Several anatomical factors can also influence the complex system of forces acting on the spinal column. One of them is height. Jacobsen et al. suggested that increasing height may induce the adverse distribution of weight and postural anomalies influencing the formation of pathological forces affecting the vertebrae, which may predispose to slip formation [7]. Both He et al. and Wang et al. reported that people with spondylolisthesis were more likely to be shorter. This relationship applied to both women and men [27,74]. A similar tendency was visible in the group analyzed by Kauppila et al. However, it concerned only subjects with a degenerative slip at two or more lumbar levels. In the case of people with spondylolisthesis at one level, similar relationships were not found [18]. A group of patients with spondylolisthesis, compared to a control group, was also characterized by a lower height in a study by Schuller et al. However, in this case, height was not statistically significant [24]. Also, Denard et al. and Fraser et al. did not find a significant correlation between height and spondylolisthesis in their observations [9,32]. Interesting conclusions were found in a study by Jacobsen et al. In their analysis, there was a relationship between the height of the patients and the presence of a slip. However, increasing age was a risk factor for spondylolisthesis both at the L4 and L5 levels [7]. There is a lot of data in the literature on the relationship between other anatomical parameters and the prevalence of spondylolisthesis. This applies in particular to the parameters defining the morphology of the appendages and joint surfaces. This feature is crucial in maintaining the stability of the spine and the appropriate distribution of loads acting on its elements because facet joints are responsible for the transmission of 35% of the static load and 33% of the dynamic load affecting the spinal column [85,86,87]. These parameters include facet joint angle (FJA), sacral slope, pelvic tilt, pelvic incidence, iliac crest, and the lumbar lordosis angle [25,29,33,57,88,89,90]. However, it is not clear whether these anomalies determine a predisposition to the formation of a slip or are only the result of remodeling from already existing degenerative changes. Currently, their role is limited mainly to the radiological description of patients affected by the problem of instability; therefore, the precise characterization of these parameters is beyond the scope of this review.

### 3.4. Age

Two important aspects include the occurrence of a predisposing factor and the duration of its action. Due to their nature, degenerative changes are usually more severe in older people, where age is associated, among others, with longer exposure to harmful loads. For this reason, many researchers consider age as the primary risk factor for degenerative spondylolisthesis. Rosenberg et al. performed an analysis of two hundred patients with degenerative spondylolisthesis and noted that this ailment is more common in patients over 50 years of age [15]. In the following years, many authors supported this thesis. Mariconda et al. showed that the aging process is directly related to the formation of spondylolisthesis [21]. Similar conclusions were found by Jacobsen et al. in their analysis carried out as part of the Copenhagen Osteoarthritis Study. The authors included data from 2506 women and 1495 men, and the age of the patients varied from 22 to 93 years. As one of the main risk factors for the development of degenerative spondylolisthesis, the authors indicated the growing age of patients. This trend concerned both women and men. Interestingly, the authors noticed the opposite trend for people over 70 years of age. However, this was probably associated with a decrease in patient survival. It has also been shown that degenerative spondylolisthesis at the L4-L5 level is very rare before the age of 50 [7]. Similar conclusions were reported in the study by Kalichman et al. The authors reported no cases of degenerative slips in patients under a certain age. For men, the limit was 40 years, while for women, it was 50 years [8]. Ferrero et al. reviewed 654 degenerative spondylolisthesis cases and found that the percentage of patients under 40 years of age was 1%. People between 40 and 60 years old constituted 26% of the cases, while for the age group over 60, the percentage was 73% [28]. A more detailed analysis of the occurrence of a slip in elderly age was presented in the work of Wang et al. In this case, the authors analyzed only patients over 65 years of age. They also showed that the risk of a slip increases with age. Its presence was observed in 23.1% of men aged 65–69 years, 25.4% aged 70–74 years, 30.7% aged 75–79 years, and 30.5% of men over 80 years of age. For women, these values were respectively: 31.0%, 34.9%, 34.9%, and 39.8% [74]. Interestingly, the work of Marty-Poumarat et al. showed a relationship between the prevalence of slip and the age of patients only in the group of people who did not receive HRT (hormone replacement therapy) or took it for less than 1 year. There was no such relationship among patients who received hormones chronically [26]. Kauppila et al. also came to interesting conclusions. They found a relationship between age and the prevalence of degenerative spondylolisthesis in patients with slippage in two or more levels, while no such relationship was found when slippage was present on one lumbar level [18]. Moreover, the observations of Enyo et al. emphasized the role of age as a risk factor for progression slip. Patients at least 60 years of age were more exposed to the progression of degenerative changes in the L4 level [87]. The influence of aging on the prevalence of spondylolisthesis was also noted in studies by other authors [4,6,9,22,27,31]. The above data indicate that age is the most frequently mentioned risk factor for the risk of slip. This may result from both longer exposure to harmful factors and the accumulation of degenerative changes. On the other hand, the results of some studies do not agree on this issue. Horikawa et al. conducted a study on the Japanese population and did not show any significant correlation between aging and the prevalence of spondylolisthesis [20]. Similar conclusions were also reported in the study by Chen et al., Aono et al., and Ishimoto et al. [23,25,33]. The final settlement of this issue requires further observations.

The decline in the quality of bone material that is common in the elderly may also be significant. Pathological values of bone mineral density (BMD) were more often demonstrated in patients with spondylolisthesis [27,74,91]. The effect of bone density changes on the severity of degenerative processes was also analyzed by Wang et al. They found that disc degeneration and space narrowing is more severe among elderly women compared to the male population. In addition, they pointed to the relationship between the severity of this type of lesion and high bone mineral density values. This concerned both vertebral BMD and hip BMD. The degenerative changes in the discs can then lead to a higher degenerative spondylolisthesis prevalence [30,92,93]. The role of other factors that could influence the quality of bone material, such as calcium supply, intake of corticosteroids, history of fractures, as well as smoking and alcohol consumption, was also not reported [4,7,9,18,33,74].

### 3.5. Ethnic Origin and Genetic Factors

Analyzing the observations made on different populations, some distinctions can be seen in the prevalence of spondylolisthesis depending on ethnicity. As mentioned previously, the data available in the literature show large discrepancies in the incidence of slips [3,4,5,6,7,8,9,10,11]. One of the reasons for this may be the ethnic differences between the studied populations. A study by Denard et al. conducted on mostly Caucasian (90%) male representatives estimated the incidence of spondylolisthesis at 31% [9]. In another study carried out on patients included in the Framingham Heart Study, Kauppila et al. showed that a degenerative slip was present in 20% of the included subjects. The Framingham Heart Study cohort was created to best match American society in terms of genealogy and ethnic makeup [18]. The analysis of individuals included in that study was also undertaken by Kalichman et al. In that case, the overall prevalence of spondylolisthesis was estimated at 13.6% [8]. Vogt et al. studied the incidence of slips in 2401 Caucasian women. They determined that degenerative spondylolisthesis concerned as many as 43.1% of the studied women. Overall, 28.9% of the cases were anterolisthesis, while 14.2% of the cases were retrolisthesis [4]. Similar observations were also found in a study on African American women. In this case, the overall prevalence of anterolisthesis was 58.3%, while retrolisthesis concerned only 4% of subjects [91]. These conclusions coincide with the observations of Dent et al., who found two times more frequent occurrence of slips among Black Durban residents. Spondylolisthesis L5-S1 or L4-L5 occurred in 8% of Bantu female representatives compared to 4% of European female representatives [14]. Studies have shown that the risk of sliding is also greater among Inuit patients [9]. A comparison of the above data with observations from an Asian population also showed significant differences. A study by Enyo et al. conducted on residents of Miyama, a mountain village in Wakayama Prefecture, in Japan, determined the overall incidence of degenerative spondylolisthesis at 10% [87]. Similar values were found in a study by Iguchi et al. In their study, the incidence was 8.7% [10]. An analysis by Horikawa et al. showed that a slip was present in 4.9% of men and 11.5% of women included in their study [20]. This is consistent with the results of Aono et al., who carried out a study on women in Japan. The authors estimated the incidence of degenerative spondylolisthesis at 12.7% [25]. Chen et al. analyzed a population of taxi drivers in Taipei that was overwhelmingly male (96%). In this case, among people over 45 with lower back pain, the prevalence of slip was 8.9% [6]. Wang et al. studied the presence of spondylolisthesis in the inhabitants of Hong Kong. During their study, de novo lesions were detected in 12.7% of women and in 12.4% of men [74]. It is also worth mentioning the observations obtained by Jacobsen et al. during their study conducted on the European population. The authors observed the presence of a slip in only 6.5% of the qualified subjects [7]. The relationship between the prevalence of spondylolisthesis and the ethnic background of patients raises the suspicion of a potential role of genetic factors in its etiology [94]. In another study, Hakim et al. investigated the severity of joint hypermobility, widely recognized as one of the etiological factors for the formation of slip, in a population of female twins. They showed that in the case of monozygous twins, there is a greater agreement regarding the prevalence of spondylolisthesis compared to dizygotic twins (60% vs. 36%). This suggests a significant role of hereditary factors in this group of subjects [95]. M.D. Ryan also focused on the incidence of a slip among twins. In his case report, he described a pair of 73-year-old monozygotic twins with degenerative spondylolisthesis at the L4-L5 levels. Interestingly, one of the siblings had a profession that involved major spinal loading throughout their life, drank little alcohol, was physically active, and did not smoke, while the other sibling had a less physical occupation, was less physically active, drank more alcohol regularly, and was a smoker. Nevertheless, both subjects had a slip. Currently, many genes are indicated that are implicated in the etiology of degenerative changes in the lumbar spine [96,97,98]. In their work, Jiang et al. analyzed the influence of some of these genes (rs1337185, rs5275, rs5277, rs7575934, rs3213718, and rs162509) on the prevalence of lumbar disc degeneration in the Chinese Han population. The results showed that the C allele of rs1337185 is a risk factor for spinal stenosis and disc herniation [99]. These conclusions raise suspicions about the potential role of other genes in the etiology of this type of change.

### 3.6. Gender and the Influence of Sex Hormones

Another feature that may affect the prevalence of degenerative spondylolisthesis is the gender of the patient. Most of the authors agree unequivocally that women are more prone to slip performance. Research conducted by Kalichman et al. in the United States suggested that, in the case of spondylolisthesis, the ratio of women to men is 3:1 (21.3% vs. 7.7%) [8]. Similar results were reported in other American studies by Kauppila et al. based on the Framingham Heart Study. In this case, de novo spondylolisthesis was reported in 25% of women and only 12% of men [18]. Congruous conclusions resulted from studies carried out in Japan. Horikawa et al. described a significant difference in the prevalence of spondylolisthesis by sex. In the case of the female group, this problem affected 11.5% of patients (37 of 323 cases), while for men, the percentage was only 4.9% (10 out of 205 cases) [20]. This was in line with data from other observations of the Japanese population. Enyo et al. demonstrated the presence of slip in 11.8% of women and 7.4% of men [87]. Jacobsen et al. noticed a similar relationship. Their study was carried out on the Danish population, for which there were 2.4–4 times more frequent occurrences of degenerative spondylolisthesis at L3 and L4 levels among women. This trend was also maintained regarding the general prevalence of DS. It concerned 8.3% of the study group of women and 2.7% of men [7]. The existence of even greater disproportions was indicated in studies by Sanderson et al. In their study, the prevalence of degenerative spondylolisthesis in the female population ranged from 16.7% to 28% (depending on the number of offspring per patient) while for men, this ratio was only 7.5% [17]. Interesting conclusions came from the observations of Iguchi et al. The authors analyzed 201 cases of degenerative spondylolisthesis in the Japanese population and showed that anterolisthesis is much more common in women, while the prevalence of retrolisthesis is similar in both sexes [10]. Analogous relationships were also observed in the works of other researchers [15,21,22,28]. Additionally, Enyo et al. estimated the risk of slip progression as being more than three times higher in women than in men [87]. However, Matsunga et al. did not note such a relationship [39]. Some authors did not show any statistically significant difference in the prevalence of spondylolisthesis by gender. He et al. found only an inconsiderable discrepancy. This type of change concerned 25% of women and 19.1% of men [27]. An even smaller disproportion was visible in the case of de novo listhesis in a study by Wang et al. (12.7% vs. 12.4%) [74]. Ischimoto et al. found no difference in the prevalence of slips between women and men in the Japanese population [33].

However, despite these deviations, the vast majority of studies indicate a greater exposure of women to the occurrence of these types of changes. The relationship between the prevalence of spondylolisthesis and the gender of patients may be related to the influence of female sex hormones on the locomotor system. In earlier years, many authors studied the influence of estrogens on tissues associated with the human musculoskeletal system [19,30,92,93,100,101,102,103,104,105,106,107]. This thesis may be confirmed by the observations comparing patients in whom the regulation of sex hormones is disturbed. This applies, inter alia, to women after surgical removal of the ovaries in whom the hormone levels are significantly lowered. Previous studies have demonstrated that bilateral oophorectomy significantly affects the functions of the skeletal and muscular systems [108,109]. Imada et al. observed Japanese women and noted that patients had a higher incidence of a slip at L4-L5 after oophorectomy. Of the 69 oophorectomized patients, the occurrence of degenerative spondylolisthesis was 3 times more frequent compared with the individuals without the operation [16]. Congruous conclusions were made by Vogt et al. on a group of African American women. The authors noticed that the slip prevalence was lower among patients after oophorectomy [5]. Interestingly, Vogt et al. previously demonstrated a lower prevalence of anterolisthesis at the L5-S1 level in women after oophorectomy. However, a similar trend was not observed in the case of retrolisthesis [4]. Imada et al. suspected that a decrease in hormone levels (as a result of oophorectomy) affects the lower elasticity of the ligament apparatus leading to a slip [16]. A similar theory was also proposed by Shitaka et al. in the etiology of hip dislocation in patients after oophorectomy [110]. This suggests a potential relationship between the deficiency of sex hormones and changes in the musculoskeletal system predisposing to the formation of a listhesis.

A decrease in sex hormone levels also occurs during menopause. However, Wang et al. and Jacobsen et al. observed no relationship between menopause age and the prevalence of spondylolisthesis [7,74]. The same relationship was evident in the results obtained by Cholewicki et al. [31]. Similar issues were discussed by Marta-Poumarat. The authors investigated the impact of taking hormone replacement therapy on postmenopausal women. They showed that the prevalence of lateral rotatory oligolisthesis (LRO) was significantly lower in the group of women taking hormones for more than 1 year than in the control group (8 vs. 30%). The authors suggested this type of treatment as a potential prevention for the occurrence and also the progression of slip [26]. Convergent conclusions were reported in a study by Vogt et al. The authors noted the protective role of hormone replacement therapy (HRT) in the occurrence of anterolisthesis. However, a similar relationship was not observed in the case of retrolisthesis [5]. Not all data available in the literature are consistent on this issue. Observations made by both He et al. and Wang et al. showed no effect of estrogen intake on the prevalence of spondylolisthesis [27,74]. Earlier studies by Vogt et al. should also be mentioned. The authors did not find a significant difference in the prevalence of anterolisthesis or retrolisthesis depending on the HRT taken. However, women taking estrogens were more prone to anterolisthesis at the L5-S1 level. In the case of retrolisthesis, an increase in the incidence of slip was twofold in comparison with women not subjected to supplementation [4]. The effectiveness of HRT application was also demonstrated in other degenerative conditions such as attenuate atherosclerosis, osteoarthritis of facet joints, and intervertebral disc degeneration [111,112].

Different levels of sex hormones also occur during pregnancy. Research by Cholewicki et al. and Sanderson and Fraser have shown that pregnancy and the number of childbirths are risk factors for the occurrence of a slide [31,32]. However, not all authors agree on this issue [7,27,74].

Hormones may also influence the tone and condition of the muscular system, which, as shown in past studies, also influences the prevalence of spondylolisthesis [74,113,114,115,116,117,118,119]. Another aspect is the degradation of articular cartilage under the influence of estrogens [107,120,121,122,123,124,125]. Ha et al. investigated the relationship between the occurrence of a slip and the increase in the expression of estrogen receptors on the surface of joints. The authors showed that a large number of this type of receptor correlates significantly with the severity of degenerative changes. They were manifested, among others, as erosion, changes in the subchondral bone, and fibrillation and architectural disorders of the cartilage surface. Significant overexpression of estrogen receptors has also been shown among patients with degenerative spondylolisthesis [44]. Roh et al. also focused on estrogen receptors in patients with degenerative spondylolisthesis. In their study, the authors demonstrated a relationship between a polymorphism of this type of receptor and the severity of slip-related symptoms [126]. A similar relationship was evident in an earlier work by Lee et al. [127].

### 3.7. Other Factors

The endocrine system is also of great importance, affecting the ligamentous and muscular apparatus, and thus contributing to the formation of spondylolisthesis. The Farfan study, based on the autopsy of 460 lumbar spines, showed that among the cases meeting the criteria of degenerative spondylolisthesis (with known medical history), as many as 43% had diabetes requiring insulin supplementation [11]. Similar conclusions were suggested in the observations of Rosenberg et al. Moreover, Anekstein et al. showed that L4-5 degenerative spondylolisthesis was significantly more common in non-diabetic patients with spinal stenosis compared to those suffering from diabetes [128]. However, most authors found no similar relationship between diabetes and slip prevalence [4,5,24,27,31]. Some authors also analyzed the influence of other comorbidities. Wang et al. checked the relationship of arterial hypertension with the presence of slip. The authors noted that high-pressure values were associated with spondylolisthesis in the female group. No similar relationship was observed in the case of chronic obstructive pulmonary disease in either men or women [74].

**Figure 1:**
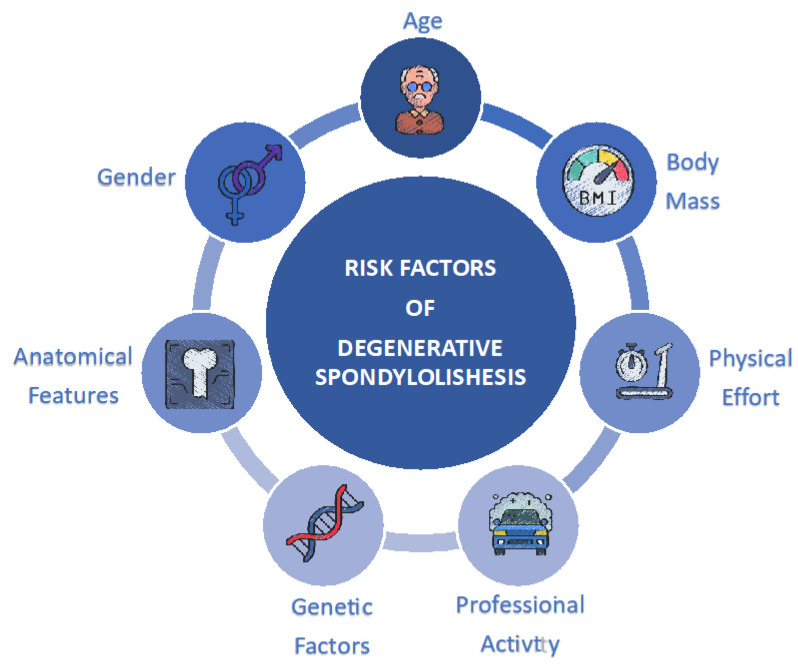
Graphic image presenting risk factors for spondylolisthesis.

A summary of the risk factors analyzed in the publications included in this review is presented in Table 2 and Figure 1.

### 3.8. The Importance of Research

Identification of the risk factors for degenerative spondylolisthesis is very important because it allows for a more detailed understanding of the pathophysiology of this type of change, which may contribute to the implementation of preventive actions in predisposed people. These preventative actions may concern lifestyle modifications, early implementation of physiotherapeutic treatments, or appropriate planning of optimal therapy in conditions potentially not related to the spine. This also applies to women in the perimenopausal age and women who have undergone gynecological operations. A full understanding of the role of hormones in the etiology of slip could help to properly regulate their endocrine balance, thus preventing listhesis. The analysis of risk factors shows that they may also affect the effectiveness of surgical treatment and the quality of life of patients with spondylolisthesis, helping to better define the need for surgery [81,82]. It could allow for earlier implementation of proper diagnostic and therapeutic procedures at a very early stage of a slip. In many cases, this would probably allow for surgery and the risk of complications to be avoided. Additional information could also enable more effective planning of the operation itself and supplement the analysis of profits and losses resulting from its implementation. After stabilization of the spine, patients are exposed to loss of stability and slip at levels adjacent to the stabilized area. Information on the occurrence of risk factors for slippage would allow for a more in-depth analysis of the requirement to perform surgery instead of using other methods of treatment.

## 4. Conclusions

There are many scientific papers identifying risk factors for the development of degenerative spondylolisthesis. These can be both population features such as age, gender, and ethnicity of study participants as well as lifestyle factors. These include proper diet and body weight, occupation, bone density, and physical activity. Along with the development of diagnostic imaging, anatomical conditions that may contribute to vertebral slip have also been analyzed. Much attention was devoted to the sagittalization of facet joints, but the potential importance of such parameters such as iliac crest, pelvic tilt, pelvic incidence, sacral slope, and lumbar lordosis was also emphasized. It should be noted that for some of the listed risk factors (age, gender, tropism of the articular surfaces), the authors mostly agree on their role in the etiology of spondylolisthesis, but for some factors, such as physical activity, the observations remain ambiguous. Further thorough research will be needed for a better understanding of this disease. Identification of risk factors for degenerative spondylolisthesis could allow for earlier implementation of proper diagnostics, appropriate prophylactic measures, and therapeutic procedures at a very early stage of a vertebral slide and thus delay or prevent surgical treatment. Additional information could also enable more effective planning of the operation. After spine stabilization, patients are potentially exposed to the risk of loss of stability and slide at levels adjacent to the stabilized area. Information on the occurrence of risk factors for slippage would allow for a more in-depth analysis of the requirement to perform surgery versus establishing a treatment path that is appropriate for a particular patient.

## Figures and Tables

**Table 1:** Characteristics of groups and criteria for inclusion and exclusion applied to the studies analyzed in this review; y—years, DS—degenerative spondylolisthesis.

Study	Study Groups	Inclusion Criteria	Exclusion Criteria:	Article Type	Main Conclusions
Dent [14]	N = 300 Age (range)—50–90 y Gender: Women—100%; Men—0%.	Group A—rural Bantu; Group B—urban Bantu; Group C—women of European origin.	Immobilization for more than a week; Systemic disease; Receiving drugs known to affect bone metabolism.	Original research	Ethnic origin has an impact on the incidence of spondylolisthesis (it is higher in a population of African women).
Rosenberg [15]	N = 200 Age (range): 44–89 y Gender: Women—79.5%; Men—20.5%.	Diagnosis of degenerative spondylolisthesis.	Patients with slipping of less than 5%	Original research	Sex, age, and diabetes are listed as predisposing risk factors.
Imada [16]	Study group: N = 210 Age (mean): DS: 58.8 ± 6.49 y; Control: 58.7 ± 6.54 y. Gender: Women—100%; Men—0%. Cohort: N =138 Gender: Women—100%; Men—0%. Age (mean): Oophorectomy: 53.8 y; Non-oophorectomy: 53.6 y.	Study Group: Women with low back pain; Diagnosis of degenerative spondylolisthesis. Controls: A total of 105 patients matched by age, gender, and occupation. Cohort: A total of 69 patients after bilateral oophorectomy, before menopause and without hormone replacement therapy; Randomly sampled comparison group of 69 non-oophorectomized patients.	None reported	Case-control study	Decreased level of sex hormones is a risk factor for degenerative spondylolisthesis.
Sanderson [17]	N = 1069 Age (mean): Men—64.1 ± 5.8 y; Parous women—63.1 ± 5.1 y; Nulliparous women—61.2 ± 6.4 y. Gender: Women—88.8%; Men—11.2%.	Lower back pain; History of spine surgery.	None reported	Original research	Pregnancy is a risk factor for the development of degenerative spondylolisthesis.
Kauppila [18]	N = 617 Females—400; Man—217.	A 25-year follow-up study in Framingham Cohort; Lumbar radiographs available.	None reported	Original research	The incidence of spondylolisthesis is associated with age.
Vogt [4]	N = 788 Age (mean): 71.5 y Gender: Women—100%; Men—0%.	White women; Aged more than 65 y; Participants of a multicenter study on osteoporotic fractures (SOFs); Living in the general community within a 25-mile radius of the clinic.	Man; African american women; Women who were institutionalized; Women after bilateral hip replacement; Inability to walk without the assistance of another person.	Cross-sectional study	Age, sex, and ethnic origin are factors predisposing for spondylolisthesis.
Vogt [19]	N = 1366 Age (mean)—71.2 ± 5.1 y Gender: Women—100%; Men—0%.	White women; Aged more than 65 y; Participants of a multicenter study on osteoporotic fractures (SOFs); Living in the general community within 25-mile radius of the clinic.	Institutionalized, bilateral hip replacement; Patients unable to walk without assistance of another person; Man; African American women.	Cross-sectional and prospective study	Elevated spinal bone mineral density is a risk factor for retrospondylolisthesis.
Iguchi [10]	N = 201 Age (mean): 64.6 y Gender: Females—115; Males—86.	Diagnosis of degenerative spondylolisthesis.	Trauma, tumors, osteoporotic compression fractures, and major systemic diseases (cerebrovascular disease, diabetes mellitus); Degenerative scoliosis of >10°; Spondylolysis and spondylolytic spondylolisthesis	Original research	Anterolisthesis is much more common in women; the prevalence of retrolisthesis is similar in both sexes.
Vogt [5]	N = 470 Age (mean)—75.1 ± 4.9 y Gender: Women—100%; Men—0%.	African American women; Aged at least 65 years.	Women who were institutionalized; Women after bilateral hip replacement; Inability to walk without the assistance of another person.	Cross-sectional epidemiological study	Ethnic origin is a risk factor for spondylolisthesis; HRT reduces risk of anterolisthesis; Diabetes is excluded as a risk factor.
Chen [6]	N = 1242 Age (mean)—44.5 ± 8.7 y Gender: Women—4%; Men—96%.	Participants in the Taxi Drivers’ Health Study (TDHS); Registered taxi driver in Taipei City for at least one year; Willing to participate; Able to read.	Isthmic spondylolisthesis.	Cross-sectional epidemiological study	Sex (female), age, high BMI, and type of exercise and occupation are associated with higher incidence of spondylolisthesis.
Horikawa [20]	N = 528 Age (mean): 70.6 y Gender: Women—61.2%; Men—38.8%.	Age ≥ 65 y; Residing in a village located in Nansei-cho, Mie prefecture, Japan.	Less than 65 y; History of trauma to the thoracolumbar spine.	Original research	Age is not a risk factor; Ethnic origin and sex (female) are associated with higher incidence of spondylolisthesis.
Jacobsen [7]	N = 4001 Age (range)—22–93 y Gender: Women—62.6%; Men—37.4%.	Participants in the Copenhagen Osteoarthritis Study; X-rays available.	History of spine surgery.	Cross-sectional epidemiological study	Professional activity did not affect incidence of spondylolisthesis; Age, BMI, and angle of lordosis are associated with spondylolisthesis in women; No correlation in men.
Mariconda [21]	N = 120 Age (mean)—57.5 ± 11.8 y Gender: Women—52%; Men—48%.	Lower back pain >1 year; MRI examination; Speak Italian.	Less than 40 y; Secondary causes of low back pain: tumor, infection, congenital anomaly, trauma, psoriasis, chronic polyarthritis, or osteoporosis; Previous back surgery.	Original research	Aging, sport, and occupation are risk factors.
Hosoe [22]	N = 250 Age (mean): DS—68.2 y; Control—46.8 y. Gender: Women—62.2%; Men—37.8%.	X-ray available. DS group: Diagnosed DS of the L5 vertebra. Control group: Random sample of 293 without changes in X-rays.	None reported	Original research; observational study	Aging and sex (female) are risk factors for the development of anterolisthesis.
Chen [23]	N = 132 Age (mean): DS—55.36 ± 5.61 y; Control: 54.90 ± 5.52 y. Gender: Women—100%; Men—0%.	Study group: First time diagnosis of lumbar spondylolisthesis; Lower back pain; Women; Age: 45–64 y. Control: No spondylolisthesis; Age and gender matched to study group.	Missing or inadequate results of radiological examinations; Combined with lumbar retrolisthesis; Posttraumatic lumbar spondylolisthesis; Received further surgical treatment.	Case-control radiographic study	Disc degeneration and increased lumbar index are the only independent factors for spondylolisthesis.
Schuller [24]	Study group: N = 49 Age (mean)—65.9 y Gender: Women—75.6%; Men- 24.4%. Control: N = 77 Age (mean)—65.5 y Gender: Women—46.8%; Men—53.2%.	Study group: Diagnosed degenerative spondylolisthesis at L4–L5. Control: Lower back pain due to moderate degenerative disc disease; No spondylolisthesis.	Aged less than 40 years; Idiopathic or degenerative scoliosis; Isthmic spondylolisthesis; Degenerative spondylolisthesis at a different level than L4–L5; Previous spinal fractures, tumors, or previous surgery.	Original research	Overweight and sagittal orientation of L4-L5 facet joints are predisposing factors for anterolisthesis.
Kalichman [8]	N = 188	Participants of the Framingham study; Diagnosis of spondylolisthesis.	None reported	Cross-sectional study	Age and sex (female) are factors predisposing to degenerative spondylolisthesis.
Aono [25]	N = 142 Age (mean): Gender: Women—100%; Men—0%.	Women; No spondylolisthesis at baseline radiographs; Baseline age of 40 years or older; Could be followed up for more than 8 years.	Males; Serious medical history or prior spinal interventions.	Prospective study	Pelvic incidence, L4 inclination, and facet sagittalization are independent factors predisposing to degenerative spondylolisthesis.
Denard [9]	N = 190 Age (mean): 74 ± 6 y Gender: Women—0%; Men—100%.	Osteoporotic Fractures in Men Male; Aged at least 65 year; Ability to walk unassisted by another person; Had at least on natural hip for femoral bone density measurement; Radiograph available.	None reported	Prospective cohort study	Age and physical activity are risk factors, whereas height, BMI, diabetes, and smoking were excluded as such.
Marty-Poumarat [26]	N = 146 HRT group—75; No HRT group—71. Age (mean): 65.8 ± 5.7 y	Women; Over 5 years after menopause. HRT group: Taking HRT for over a year. No HRT group: Not taking HRT or taking HRT for less than a year.	No total body bone density measurements; Having X-rays that were not good enough to allow the correct measurement of spine Indices; History of spinal surgery, vertebral fractures, and lumbo-sacral transitional vertebra.	Original research	HRT decreases risk of degenerative spondylolisthesis in a population of women; Aging is a risk factor.
He [27]	N = 3990 Age. mean: Women—72,6 y; Men—77,4 y. Gender Women—50%; Men—50%.	Participants of survey for osteoporotic fractures in Hong Kong; Aged 65 years or above; Living in community dwelling; Ability to walk without assistance; Potential to survive the duration of a primary study based on their general medical health; Radiograph available.	Bilateral hip replacement; Malignancy; Previous lumbar spine surgery.	Prospective population-based study	Daily exercise plays a protective role indeveloping spondylolisthesis; High BMI, short height, age, and high BMD are associated with a higher prevalence of spondylolisthesis; Sex, level of estrogen, number of childbirths and diabetes were excluded as risk factors.
Ferrero [28]	N = 654 Age (mean): 67.3 ± 10.6 y Gender: Women—72%; Men—28%.	Aged 18 years or above; Surgical treatment for one or two levels lumbar DS.	Coronal Cobb angle greater than 10; Previous spinal surgery; Pelvis or joint diseases such as severe osteoarthritis in the lower limbs, tumor, or fracture of the pelvis or lower limbs.	Retrospective multicenter study of prospectively collected data	High pelvic incidence, age, and female gender are risk factors for developing degenerative spondylolisthesis.
Enyo [29]	N = 200 Gender: Women—59.5%; Men—40.5%.	Residents of Miyama, in Wakayama Prefecture, Japan; Measurement of bone mineral density (BMD); Radiograph available.	None reported	Original research	Ethnic origin, facet sagittalization, and female gender have and impact on developing degenerative spondylolisthesis.
Wang [30]	N = 3065 Age (mean): Women—75.7 y; Men—75.5 y. Gender: Women—50.4%; Men—49.6%.	Participants of survey for osteoporotic fractures in Hong Kong; Aged 65 years or above; Living in community dwelling; Ability to walk without assistance; Potential to survive the duration of a primary study based on their general medical health.	Bilateral hip replacement; Malignancy; Previous lumbar spine surgery.	Longitudinal follow-up study	Professional activity, gender, and level of estrogen do not influence the prevalence of spondylolisthesis; Physical activity has a protective role; Short height, age, high BMI, high BMD, and high values of blood pressure in women are associated with higher prevalence of spondylolisthesis.
Cholewicki [31]	N = 322 DS—149; Controls—173. Age (range)—40–80 y Gender: Women—100%; Men—0%.	Diagnosed DS with minimum of 5% anterior slip measured in the lumbar region.	Previous spinal surgery; Traumatic injury of the spine; Not being independently ambulatory.	Case-control study	Pregnancy, number of childbirths, and histerectomy are risk factors for degenerative spondylolisthesis.
Fraser [32]	N = 205 Age (mean): 68.3 y Gender: Women—52%; Men—48%.	Aged at least 50 y.	Previous lumbar surgery; Spinal infection, neoplasm, or a fracture.	Prospective cross-sectional cohort study	Weakness of abdominal muscles, pregnancy, and number of childbirths are risk factors associated with a higher prevalence of spondylolisthesis.
Ishimoto [33]	N = 722 Spondylolisthesis—117; Control—605. Age (mean): 70.1 y Gender: Women—66%; Men—34%.	Participants of Wakayama Spine Study (WSS); Radiograph available.	The impossibility of performing an MRI examination; Previous lumbar operation.	Cross-sectional observational study	Occupational driving is a risk factor, whereas daily exercise reduces the risk of developing spondylolisthesis. No correlation with age was found.

**Table 2:** The influence of individual factors on the prevalence of spondylolisthesis. +—positive impact, −—negative impact, +/− ambiguous impact, BMI—body mass index, BMD—bone mineral density, HRT—hormone replacement therapy.

No.	Study	n	Age	BMI	BMD	Gender	Height	Ehtnic Origin	Physical Activity	Diabetes	Smoking	Pregnancy Status	HRT	Opherectomy
1	Dent [14]	300						+						
2	Rosenberg [15]	200	+			+		+		+				
3	Imada 109]	210												+
4	Sanderson [17]	1069				+						+		
5	Kauppila [18]	617	−	+/−		+	-				−			
6	Vogt [4]	788	+							−	−		+	+/−
7	Vogt [19]	1400			+			+						
8	Iguchi [10]	3259				+								
9	Vogt [5]	481						+		−			+	+
10	Chen [6]	1242	+	+					+					
11	Horikawa [20]	528	−			+		+						
12	Jacobsen [7]	4001	+	+/−		+	+/−		−		−	−		
13	Mariconda [21]	120	+			+			+					
14	Hosoe [22]	250	+			+								
15	Chen [23]	132	−											
16	Kalichman [8]	188	+			+								
17	Aono [25]	142	−	−										
18	Denard [9]	190	+	−			−		+	−	−			
19	Schuller [24]	126		+			+							
20	Marty-Poumarat [26]	147	+										+	
21	He [27]	3990	+	+	+	−	+		−	−	−	−	−	
22	Ferrero [28]	654	+			+								
23	Enyo [29]	200	+			+								
24	Wang [30]	3065	+	+	+	−	+		−	−	−	−	−	
25	Cholewicki [31]	322	+	+		+						+		
26	Fraser [32]	205		−			−					+		
27	Ishimoto [33]	722	−	−		−			+		−			

## References

[1] Errico T.J., Lonner B.S., Moulton A.W. (2009). Surgical Management of Spinal Deformities.

[2] Shamrock A.G., Donnally C.J., Varacallo M. (2019). Lumbar Spondylolysis and Spondylolisthesis.

[3] DeVine J.G., Schenk-Kisser J.M., Skelly A.C. (2012). Risk factors for degenerative spondylolisthesis: A systematic review. Evid. Based Spine-Care J..

[4] Vogt M.T., Rubin D., Valentin R.S., Palermo L., Donaldson W.F., Nevitt M., Cauley J.A. (1998). Lumbar Olisthesis and Lower Back Symptoms in Elderly White Women. Spine.

[5] Vogt M.T., Rubin D.A., Palermo L., Christianson L., Kang J.D., Nevitt M.C., Cauley J.A. (2003). Lumbar spine listhesis in older African American women. Spine J..

[6] Chen J.C., Chan W.P., Katz J.N., Chang W., Christiani D. (2004). Occupational and personal factors associated with acquired lumbar spondylolisthesis of urban taxi drivers. Occup. Environ Med..

[7] Jacobsen S., Sonne-Holm S., Rovsing H., Monrad H., Gebuhr P. (2007). Degenerative lumbar spondylolisthesis: An epidemiological perspective: The Copenhagen Osteoarthritis Study. Spine.

[8] Kalichman L., Kim D.H., Li L., Guermazi A., Berkin V., Hunter D.J. (2009). Spondylolysis and spondylolisthesis: Prevalence and association with low back pain in the adult community-based population. Spine.

[9] Denard P.J., Holton K.F., Miller J., Fink H.A., Kado D.M., Yoo J.U., Marshall L.M. (2010). Lumbar spondylolisthesis among elderly men: Prevalence, correlates and progression. Spine.

[10] Iguchi T., Wakami T., Kurihara A., Kasahara K., Yoshiya S., Nishida K. (2002). Lumbar Multilevel Degenerative Spondylolisthesis: Radiological Evaluation and Factors Related to Anterolisthesis and Retrolisthesis. J. Spinal Disord. Tech..

[11] Farfan H.F. (1980). The pathological anatomy of degenerative spondylolisthesis. A cadaver study. Spine.

[12] Wiltse L.L. (1981). Classification, Terminology and Measurements in Spondylolisthesis. Iowa Orthop. J..

[13] Syrmou E., Tsitsopoulos P.P., Marinopoulos D., Tsonidis C., Anagnostopoulos I., Tsitsopoulos P.D. (2010). Spondylolysis: A review and reappraisal. Hippokratia.

[14] Dent C.E., Engelbrecht H.E., Godfrey R.C. (1968). Osteoporosis of Lumbar Vertebrae and Calcification of Abdominal Aorta in Women Living in Durban. BMJ.

[15] Rosenberg N.J. (1975). Degenerative spondylolisthesis. Predisposing factors. J. Bone Jt. Surg. Am..

[16] Imada K., Matsui H., Tsuji H. (1995). Oophorectomy predisposes to degenerative spondylolisthesis. J. Bone Jt. Surg. Br..

[17] Sanderson P.L., Fraser R.D. (1996). The influence of pregnancy on the development of degenerative spondylolisthesis. J. Bone Jt. Surg. Br..

[18] Kauppila L.I., Eustace S., Kiel D.P., Felson D.T., Wright A.M. (1998). Degenerative Displacement of Lumbar Vertebrae. A 25-year follow-up study in Framingham. Spine.

[19] Yamamuro T., Hama H., Takeda T., Shikata J., Sanada H. (1977). Biomechanical and hormonal factors in the etiology of congenital dislocation of the hip joint. Int. Orthop..

[20] Horikawa K., Kasai Y., Yamakawa T., Sudo A., Uchida A. (2006). Prevalence of Osteoarthritis, Osteoporotic Vertebral Fractures, and Spondylolisthesis among the Elderly in a Japanese Village. J. Orthop. Surg..

[21] Mariconda M., Galasso O., Imbimbo L., Lotti G., Milano C. (2007). Relationship between alterations of the lumbar spine, visualized with magnetic resonance imaging, and occupational variables. Eur. Spine J..

[22] Hosoe H., Ohmori K. (2008). Degenerative lumbosacral spondylolisthesis: Possible factors which predispose the fifth lumbar vertebra to slip. J. Bone Jt. Surg. Br..

[23] Chen I.-R., Wei T.-S. (2009). Disc Height and Lumbar Index as Independent Predictors of Degenerative Spondylolisthesis in Middle-Aged Women with Low Back Pain. Spine.

[24] Schuller S., Charles Y.P., Steib J.-P. (2010). Sagittal spinopelvic alignment and body mass index in patients with degenerative spondylolisthesis. Eur. Spine J..

[25] Aono K., Kobayashi T., Jimbo S., Atsuta Y., Matsuno T. (2010). Radiographic Analysis of Newly Developed Degenerative Spondylolisthesis in a Mean Twelve-Year Prospective Study. Spine.

[26] Marty-Poumarat C., Ostertag A., Baudoin C., Marpeau M., de Vernejoul M.-C., Cohen-Solal M. (2012). Does hormone replacement therapy prevent lateral rotatory spondylolisthesis in postmenopausal women?. Eur. Spine J..

[27] He L.-C., Wang Y.-X.J., Gong J.-S., Griffith J.F., Zeng X.-J., Kwok A.W., Leung J.C., Kwok T., Ahuja A.T., Leung P.C. (2014). Prevalence and risk factors of lumbar spondylolisthesis in elderly Chinese men and women. Eur. Radiol..

[28] Ferrero E., Ould-Slimane M., Gille O., Guigui P., French Spine Society (SFCR) (2015). Sagittal spinopelvic alignment in 654 degenerative spondylolisthesis. Eur. Spine J..

[29] Enyo Y., Yoshimura N., Yamada H., Hashizume H., Yoshida M. (2015). Radiographic natural course of lumbar degenerative spondylolisthesis and its risk factors related to the progression and onset in a 15-year community-based cohort study: The Miyama study. J. Orthop. Sci..

[30] Wang Y.-X.J., Griffith J.F., Zeng X.-J., Deng M., Kwok A.W.L., Leung J.C.S., Ahuja A.T., Kwok T., Leung P.C. (2013). Prevalence and Sex Difference of Lumbar Disc Space Narrowing in Elderly Chinese Men and Women: Osteoporotic Fractures in Men (Hong Kong) and Osteoporotic Fractures in Women (Hong Kong) Studies. Arthritis Rheum..

[31] Cholewicki J., Lee A.S., Popovich J.M., Mysliwiec L.W., Winkelpleck M.D., Flood J.N., Pathak P.K., Kaaikala K.H., Reeves N.P., Kothe R. (2017). Degenerative Spondylolisthesis Is Related to Multiparity and Hysterectomies in Older Women. Spine.

[32] Fraser R.D., Brooks F., Dalzell K. (2018). Degenerative spondylolisthesis: A prospective cross-sectional cohort study on the role of weakened anterior abdominal musculature on causation. Eur. Spine J..

[33] Ishimoto Y., Cooper C., Ntani G., Yamada H., Hashizume H., Nagata K., Muraki S., Tanaka S., Yoshida M., Yoshimura N. (2019). Is radiographic lumbar spondylolisthesis associated with occupational exposures? Findings from a nested case control study within the Wakayama spine study. BMC Musculoskelet. Disord..

[34] Junghanns H. (1931). Spondylolisthesen ohne spalt im Zwishengelenkstuck. Arch. Orthop. Unfallchir..

[35] Macnab I. (1950). Spondylolisthesis with an intact neural arch; the so-called pseudo-spondylolisthesis. J. Bone Jt. Surg. Br..

[36] Newman P.H., Stone K.H. (1963). The etiology of spondylolisthesis. J. Bone Jt. Surg. Br..

[37] Fitzgerald J., Newman P.H. (1976). Degenerative spondylolisthesis. J. Bone Jt. Surg. Br..

[38] Kalichman L., Hunter D.J. (2008). Degenerative lumbar spondylolisthesis: Anatomy, biomechanics and risk factors. J. Back Musculoskelet. Rehabil..

[39] Matsunaga S., Sakou T., Morizono Y., Masuda A., Demirtas A.M. (1990). Natural History of Degenerative Spondylolisthesis: Pathogenesis and natural course of the slippage. Spine.

[40] Grobler L.J., Robertson P.A., Novotny J.E., Pope M.H. (1993). Etiology of spondylolisthesis. Assessment of the role played by lumbar facet joint morphology. Spine.

[41] Kirkaldy-Willis W.H., Farfan H.F. (1982). Instability of the lumbar spine. Clin. Orthop..

[42] Taillard W.F. (1976). Etiology of spondylolisthesis. Clin. Orthop..

[43] Farfan H.F., Sullivan J.B. (1969). The relation of facet orientation to intervertebral disc failure. Can. J. Surg..

[44] Ha K.-Y., Chang C.-H., Kim K.-W., Kim Y.-S., Na K.-H., Lee J.-S. (2005). Expression of Estrogen Receptor of the Facet Joints in Degenerative Spondylolisthesis. Spine.

[45] Sengupta D.K., Herkowitz H.N. (2005). Degenerative spondylolisthesis: Review of current trends and controversies. Spine.

[46] Fujiwara A., Tamai K., An H.S., Kurihashi A., Lim T.-H., Yoshida H., Saotome K. (2000). The Relationship Between Disc Degeneration, Facet Joint Osteoarthritis, and Stability of the Degenerative Lumbar Spine. J. Spinal Disord..

[47] Ito S., Yamada Y., Tuboi S., Muro T. (1990). Specific pattern of ruptured annulus fibrosus in lumbar degenerative spondylolisthesis. Neuroradiology.

[48] Frymoyer J.W. (1994). Degenerative Spondylolisthesis: Diagnosis and Treatment. J. Am. Acad. Orthop. Surg..

[49] Gao F., Hou D., Zhao B., Sun X., Sun H., Li N., Guo L., Liu C. (2012). The pedicle-facet angle and tropism in the sagittal plane in degenerative spondylolisthesis: A computed tomography study using multiplanar reformations techniques. J. Spinal Disord Tech..

[50] Inoue S., Watanabe T., Goto S., Takahashi K., Takata K., Sho E. (1988). Degenerative spondylolisthesis. Pathophysiology and results of anterior interbody fusion. Clin. Orthop..

[51] Sato K., Wakamatsu E., Yoshizumi A., Watanabe N., Irei O. (1989). The configuration of the laminas and facet joints in degenerative spondylolisthesis: A clinicoradiologic study. Spine.

[52] Cinotti G., Postacchini F., Fassari F., Urso S. (1997). Predisposing factors in degenerative spondylolisthesis: A radiographic and CT study. Int Orthop..

[53] Berlemann U., Jeszenszky D.J., Buhler D.W., Harms J. (1998). Facet joint remodeling in degenerative spondylolisthesis: An investi-gation of joint orientation and tropism. Eur. Spine J..

[54] Love T.W., Fagan A.B., Fraser R.D. (1999). Degenerative spondylolisthesis. Developmental or acquired?. J. Bone Jt. Surg. Br..

[55] Aihara T., Takahashi K., Yamagata M., Moriya H., Tamaki T. (2000). Biomechanical functions of the iliolumbar ligament in L5 spondylolysis. J. Orthop. Sci..

[56] Bird H.A., Eastmond C.J., Hudson A., Wright V. (1980). Is generalized joint laxity a factor in spondylolisthesis?. Scand. J. Rheumatol..

[57] Kalichman L., Hodges P., Li L., Guermazi A., Hunter D.J. (2009). Changes in paraspinal muscles and their association with low back pain and spinal degeneration: CT study. Eur. Spine J..

[58] Izzo R., Guarnieri G., Guglielmi G., Muto M. (2013). Biomechanics of the spine. Part I: Spinal stability. Eur. J. Radiol..

[59] Lai Q., Gao T., Lv X., Liu X., Wan Z., Dai M., Zhang B., Nie T. (2018). Correlation between the sagittal spinopelvic alignment and degenerative lumbar spondylolisthesis: A retrospective study. BMC Musculoskelet. Disord..

[60] Lindgren K.A., Sihvonen T., Leino E., Pitkänen M., Manninen H. (1993). Exercise therapy effects on functional radiographic findings and segmental electromyographic activity in lumbar spine instability. Arch. Phys. Med. Rehabil..

[61] Sihvonen T., Partanen J., Hanninen O., Soimakallio S. (1991). Electric behaviour of low back muscles during lumbar pelvic rhythm in low back pain patients and healthy controls. Arch. Phys. Med. Rehabil..

[62] Guo M., Kong C., Sun S., Sun X., Li X., Lu S. (2019). Predictors of L4−L5 Degenerative Lumbar Spondylolisthesis: L4 Inclination Angle and Facet Joint Angle. World Neurosurg..

[63] Stokes I.A., Gardner-Morse M. (1995). Stability increase of the lumbar spine with different muscle groups: A biomechanical in vitro study. Spine.

[64] Hung Y.J., Shih T., Chen B., Hwang Y.H., Ma L.P., Huang W.C., Liou S.H., Ho I.K., Guo Y.L. (2014). The dose response rela-tionship between cumulative lifting load and lumbar disk degeneration based on magnetic resonance imaging findings. Phys Ther..

[65] Cannata F., Vadalà G., Ambrosio L., Fallucca S., Napoli N., Papalia R., Pozzilli P., Denaro V. (2019). Intervertebral disc degeneration: A focus on obesity and type 2 diabetes. Diabetes Metab. Res. Rev..

[66] Videman T., Battié M.C., Parent E., Gibbons L.E., Vainio P., Kaprio J. (2008). Progression and determinants of quantitative magnetic resonance imaging measures of lumbar disc degeneration: A five-year follow-up of adult male monozygotic twins. Spine.

[67] Rossi F. (1978). Spondylolysis, spondylolisthesis and sports. J. Sports Med. Phys. Fit..

[68] Jakab G. (1989). 6 Occupational spondylolysis and spondylolisthesis. Baillieres Clin. Rheumatol..

[69] Muschik M., Hahnel H., Robinson P.N., Perka C., Muschik C. (1996). Competitive sports and the progression of spondylolisthesis. J. Pediatr. Orthop..

[70] Beutler W.J., Fredrickson B.E., Murtland A., Sweeney C.A., Grant W.D., Baker D. (2003). The natural history of spondylolysis and spondylolisthesis: 45-year follow-up evaluation. Spine.

[71] Ishihara H., Tsuji H., Hirano N., Ohshima H., Terahata N. (1992). Effects of Continuous Quantitative Vibration on Rheologic and Biological Behaviors of the Intervertebral Disc. Spine.

[72] Jensen A., Jepsen J.R. (2014). Vibration on board and health effects. Int. Marit. Health.

[73] Vallone M., Bono F., Quendler E., Febo P., Catania P. (2016). Risk exposure to vibration and noise in the use of agricultural track-laying tractors. Ann. Agric. Environ. Med..

[74] Wáng Y.X.J.M., Deng M.M., Griffith J.F.M., Kwok A.W., Leung J.C.M., Ahuja A.T.M., Kwok T.M., Leung P.C.M. (2016). Lumbar Spondylolisthesis Progression and De Novo Spondylolisthesis in Elderly Chinese Men and Women: A Year-4 Follow-up Study. Spine.

[75] Farfan H.F., Osteria V., Lamy C. (1976). The mechanical etiology of spondylolysis and spondylolisthesis. Clin. Orthop. Relat. Res..

[76] Tassanawipas W., Chansriwong P., Mokkhavesa S. (2005). The orientation of facet joints and transverse articular dimension in degenerative spondylolisthesis. J. Med. Assoc. Thail..

[77] Toyone T., Ozawa T., Kamikawa K., Watanabe A., Matsuki K., Yamashita T., Wada Y. (2009). Facet joint orientation difference between cephalad and caudal portions: A possible cause of degenerative spondylolisthesis. Spine.

[78] Dai F., Belfer I., Schwartz C.E., Banco R., Martha J.F., Tighioughart H., Tromanhauser S.G., Jenis L.G., Kim D.H. (2010). Association of catechol-O-methyltransferase genetic variants with outcome in patients undergoing surgical treatment for lumbar degenerative disc disease. Spine J..

[79] Smorgick Y., Mirovsky Y., Fischgrund J.S., Baker K.C., Gelfer Y., Anekstein Y. (2014). Radiographic Predisposing Factors for Degenerative Spondylolisthesis. Orthopedics.

[80] Sasagawa T., Nakamura T. (2016). Associated Factors for Lumbar Degenerative Spondylolisthesis in Japanese Patients with Osteoarthritis of the Hip: A Radiographic Study. Asian Spine J..

[81] Epstein N.E., Epstein J.A., Carras R., Lavine L.S. (1983). Degenerative Spondylolisthesis with an Intact Neural Arch: A Review of 60 Cases with an Analysis of Clinical Findings and the Development of Surgical Management. Neurosurgery.

[82] Rihn J.A., Radcliff K., Hilibrand A.S., Anderson D.T., Zhao W., Lurie J., Vaccaro A.R., Freedman M.K., Albert T.J., Weinstein J.N. (2012). Does Obesity Affect Outcomes of Treatment for Lumbar Stenosis and Degenerative Spondylolisthesis? Analysis of the Spine Patient Outcomes Research Trial (SPORT). Spine.

[83] Simmonds A.M., Rampersaud Y.R., Dvorak M.F., Dea N., Melnyk A.D., Fisher C.G. (2015). Defining the inherent stability of degenerative spondylolisthesis: A systematic review. J. Neurosurg. Spine.

[84] Evans N., McCarthy M. (2018). Management of symptomatic degenerative low-grade lumbar spondylolisthesis. EFORT Open Rev..

[85] Schmidt H., Heuer F., Wilke H.-J. (2008). Interaction Between Finite Helical Axes and Facet Joint Forces Under Combined Loading. Spine.

[86] Lorenz M., Patwardhan A., Vanderby R. (1983). Load-Bearing Characteristics of Lumbar Facets in Normal and Surgically Altered Spinal Segments. Spine.

[87] Maciejczak A., Jabłońska-Sudoł K. (2016). Correlation between correction of pelvic balance and clinical outcomes in mid- and low-grade adult isthmic spondylolisthesis. Eur. Spine J..

[88] Boden S.D., Riew K.D., Yamaguchi K., Branch T.P., Schellinger D., Wiesel S.W. (1996). Orientation of the lumbar facet joints: Association with degenerative disc disease. J. Bone Jt. Surg. Am..

[89] Chen Q., Cao L., Bian C., Wang H.-R., Lin H., Li X.-L., Jiang Y.-Q., Dong J. (2017). Degenerative Spondylolisthesis in the Fifth Lumbar Vertebra and Radiographic Parameters: A Correlation Analysis. Clin. Spine Surg..

[90] Barrey C., Jund J., Perrin G., Roussouly P. (2007). Spinopelvic alignment of patients with degenerative spondylolisthesis. Neurosurgery.

[91] Vogt M.T., Rubin D.A., San Valentin R., Palermo L., Kang J.D., Donaldson W.F., Nevitt M., Cauley J.A. (1999). Degenerative lumbar listhesis and bone mineral density in elderly women. The study of osteoporotic fractures. Spine.

[92] Wang Y.-X.J., Griffith J.F. (2010). Effect of Menopause on Lumbar Disk Degeneration: Potential Etiology. Radiology.

[93] Wang Y.-X.J., Griffith J.F., Ma H.T., Kwok A.W.L., Leung J.C.S., Yeung D.K.W., Ahuja A.T., Leung P.C. (2010). Relationship between gender, bone mineral density, and disc degeneration in the lumbar spine: A study in elderly subjects using an eight-level MRI-based disc degeneration grading system. Osteoporos. Int..

[94] Tower S.S., Pratt W.B. (1990). Spondylolysis and associated spondylolisthesis in Eskimo and Athabascan populations. Clin. Orthop. Relat. Res..

[95] Hakim A.J., Cherkas L.F., Grahame R., Spector T.D., MacGregor A.J. (2004). The genetic epidemiology ofjoint hypermobility: Apopulation studyoffemale twins. Arthritis Rheum..

[96] Eskola P.J., Lemmelä S., Kjaer P., Solovieva S., Männikkö M., Tommerup N., Lind-Thomsen A., Husgafvel-Pursiainen K., Cheung K.M.C., Chan D. (2012). Genetic Association Studies in Lumbar Disc Degeneration: A Systematic Review. PLoS ONE.

[97] Rajasekaran S., Kanna R.M., Senthil N., Raveendran M., Ranjani V., Cheung K.M.C., Chan D., Kao P.Y.P., Yee A., Shetty A.P. (2014). Genetic susceptibility of lumbar degenerative disc disease in young Indian adults. Eur. Spine J..

[98] Chan D., Song Y., Sham P., Cheung K.M. (2006). Genetics of disc degeneration. Eur. Spine J..

[99] Jiang H., Yang Q., Jiang J., Zhan X., Xiao Z. (2017). Association between *COL11A1* (rs1337185) and *ADAMTS5* (rs162509) gene polymorphisms and lumbar spine pathologies in Chinese Han population: An observational study. BMJ Open.

[100] Liu S.H., Al-Shaikh R., Yang R.-S., Nelson S.D., Soleiman N., Finerman G.A.M., Lane J.M., Panossian V. (1996). Primary immunolocalization of estrogen and progesterone target cells in the human anterior cruciate ligament. J. Orthop. Res..

[101] Ushiyama T., Inoue K., Nishioka J. (1995). Expression of estrogen receptor related protein (p29) and estradiol binding in human arthritic synovium. J. Rheumatol..

[102] Sievert L.L., Saliba M., Reher D., Sahel A., Hoyer D., Deeb M., Obermeyer C.M. (2008). The medical management of menopause: A four-country comparison care in urban areas. Maturitas.

[103] Takahashi T.A., Johnson K.M. (2015). Menopause. Med. Clin. N. Am..

[104] Wang Y.X. (2015). Post-menopausal Chinese women have accelerated lumbar disc degeneration compared with Chinese men. J. Or-thop. Translat..

[105] Akeda K., Yamada T., Inoue N., Nishimura A., Sudo A. (2015). Risk factors for lumbar intervertebral disc height narrowing: A population-based longitudinal study in the elderly. BMC Musculoskelet. Disord..

[106] Dodge H.J., Mikkelsen W.M., Duff I.F. (1970). Age-sex specific prevalence of radiographic abnormalities of the joints of the hands, wrists and cervical spine of adult residents of the Tecumseh, Michigan, Community Health Study area, 1962–1965. J. Chronic Dis..

[107] Srikanth V.K., Fryer J.L., Zhai G., Winzenberg T.M., Hosmer D., Jones G. (2005). A meta-analysis of sex differences prevalence, incidence and severity of osteoarthritis. Osteoarthr. Cartil..

[108] Min D., Xiang W.Y., Griffith J.F., Gang L., Ahuja A.T., Poon W.S. (2012). Characteristics of rat lumbar vertebral body bone mineral density and differential segmental responses to sex hormone. Biomed. Environ. Sci..

[109] Wang Y.X., Griffith J.F., Deng M., Yeung D.K., Yuan J. (2015). Rapid increase in marrow fat content and decrease in marrow per-fusion in lumbar vertebra following bilateral oophorectomy: An MR imaging-based prospective longitudinal study. Korean J. Radiol..

[110] Shikata J., Sanada H., Yamamuro T., Takeda T. (1979). Experimental Studies of the Elastic Fiber of the Capsular Ligament. Connect. Tissue Res..

[111] Baron Y.M., Brincat M.P., Galea R., Calleja N. (2005). Intervertebral disc height in treated and untreated overweight post-menopausal women. Hum. Reprod..

[112] Tostes R., Nigro D., Fortes Z., Carvalho M. (2003). Effects of estrogen on the vascular system. Braz. J. Med. Biol. Res..

[113] Kobayashi T., Chiba H., Jimbo S., Senoo I., Shimizu M., Atsuta Y., Ito H., Sugisawa H., Sugawara T., Habaguchi T. (2016). Clinical, physical, and radiographic analyses of lumbar degenerative kyphosis and spondylolisthesis among community-based cohort. Eur. Spine J..

[114] Skelton D.A., Phillips S.K., Bruce S.A., Naylor C.H., Woledge R.C. (1999). Hormone replacement therapy increases isometric muscle strength of adductor pollicis in post-menopausal women. Clin. Sci..

[115] Phillips S.K., Rook K.M., Siddle N.C., Bruce S.A., Woledge R.C. (1993). Muscle weakness in women occurs at an earlier age than in men, but strength is preserved by hormone replacement therapy. Clin. Sci..

[116] Taaffe D.R., Sipila S., Cheng S., Puolakka J., Toivanen J., Suominen H. (2005). The effect of hormone replacement therapy and/or exercise on skeletal muscle attenuation in post-menopausal women: A yearlong intervention. Clin. Physiol. Funct. Imaging.

[117] Preisinger E., Alacamlioglu Y., Saradeth T., Resch K.L., Holzer G., Metka M. (1995). Forearm bone-density and grip strength in women after menopause, with and without estrogen replacement therapy. Maturitas.

[118] Naessen T., Lindmark B., Larsen H.C. (1997). Better postural balance in elderly women receiving estrogens. Am. J. Obstet. Gynecol..

[119] Marshall S.A., Senadheera S.N., Parry L.J., Girling J.E. (2017). The Role of Relaxin in Normal and Abnormal Uterine Function During the Menstrual Cycle and Early Pregnancy. Reprod. Sci..

[120] Tsai C.L., Liu T.K. (1993). Estradiol-induced knee osteoarthrosis in ovariectomized rabbits. Clin. Orthop..

[121] Felson D.T., Nevitt M.C. (1998). The effects of estrogen on osteoarthritis. Curr. Opin. Rheumatol..

[122] Nordin E.C., Polley K.J. (1987). Metabolic consequences of the menopause: A cross-sectional, longitudinal, and intervention study on 557 normal postmenopausal women. Calcif. Tissue Int..

[123] Spector T.D., Perry L.A., Jubb R.W. (1991). Endogenous sex steroid levels in women with generalized osteoarthritis. Clin. Rheumatol..

[124] Tsai C.-L., Liu T.-K. (1992). Up-regulation of estrogen receptors in rabbit osteoarthritic cartilage. Life Sci..

[125] Tsai C., Liu T., Chen T. (1992). Estrogen and osteoarthritis: A study of synovial estradiol and estradiol receptor binding in human osteoarthritic knees. Biochem. Biophys. Res. Commun..

[126] Roh H.L., Lee J.S., Suh K.T., Kim J.I., Lee H.S., Goh T.S., Park S.H. (2013). Association Between Estrogen Receptor Gene Polymorphism and Back Pain Intensity in Female Patients with Degenerative Lumbar Spondylolisthesis. J. Spinal Disord. Tech..

[127] Lee J.S., Suh K.T., Kim J.I., Lim J.M., Goh T.S. (2011). Association of Estrogen Receptor Gene Polymorphism in Patients with Degenerative Lumbar Spondylolisthesise. J. Korean Neurosurg. Soc..

[128] Anekstein Y., Smorgick Y., Lotan R., Agar G., Shalmon E., Floman Y., Mirovsky Y. (2010). Diabetes mellitus as a risk factor for the development of lumbar spinal stenosis. Isr. Med. Assoc. J..

